# Vital Signs Predict Rapid-Response Team Activation Within Twelve Hours of Emergency Department Admission

**DOI:** 10.5811/westjem.2016.2.28501

**Published:** 2016-04-26

**Authors:** James M. Walston, Daniel Cabrera, Shawna D. Bellew, Marc N. Olive, Christine M. Lohse, M. Fernanda Bellolio

**Affiliations:** * Mayo Medical School, Rochester, Minnesota; † Department of Emergency Medicine, Mayo Clinic, Rochester, Minnesota; ‡ Department of Health Sciences Research, Division of Biostatistics, Mayo Clinic, Rochester, Minnesota; § Robert D. and Patricia E. Kern Center for the Science of Health Care Delivery, Mayo Clinic, Rochester, Minnesota

## Abstract

**Introduction:**

Rapid-response teams (RRTs) are interdisciplinary groups created to rapidly assess and treat patients with unexpected clinical deterioration marked by decline in vital signs. Traditionally emergency department (ED) disposition is partially based on the patients’ vital signs (VS) at the time of hospital admission. We aimed to identify which patients will have RRT activation within 12 hours of admission based on their ED VS, and if their outcomes differed.

**Methods:**

We conducted a case-control study of patients presenting from January 2009 to December 2012 to a tertiary ED who subsequently had RRT activations within 12 hours of admission (early RRT activations). The medical records of patients 18 years and older admitted to a non-intensive care unit (ICU) setting were reviewed to obtain VS at the time of ED arrival and departure, age, gender and diagnoses. Controls were matched 1:1 on age, gender, and diagnosis. We evaluated VS using cut points (lowest 10%, middle 80% and highest 10%) based on the distribution of VS for all patients. Our study adheres to the STROBE (Strengthening the Reporting of *Observational Studies* in Epidemiology) guidelines for reporting observational studies.

**Results:**

A total of 948 patients were included (474 cases and 474 controls). Patients who had RRT activations were more likely to be tachycardic (odds ratio [OR] 2.02, 95% CI [1.25–3.27]), tachypneic (OR 2.92, 95% CI [1.73–4.92]), and had lower oxygen saturations (OR 2.25, 95% CI [1.42–3.56]) upon arrival to the ED. Patients who had RRT activations were more likely to be tachycardic at the time of disposition from the ED (OR 2.76, 95% CI [1.65–4.60]), more likely to have extremes of systolic blood pressure (BP) (OR 1.72, 95% CI [1.08–2.72] for low BP and OR 1.82, 95% CI [1.19–2.80] for high BP), higher respiratory rate (OR 4.15, 95% CI [2.44–7.07]) and lower oxygen saturation (OR 2.29, 95% CI [1.43–3.67]). Early RRT activation was associated with increased healthcare utilization and worse outcomes including increased rates of ICU admission within 72 hours (OR 38.49, 95%CI [19.03–77.87]), invasive interventions (OR 5.49, 95%CI [3.82–7.89]), mortality at 72 hours (OR 4.24, 95%CI [1.60–11.24]), and mortality at one month (OR 4.02, 95%CI [2.44–6.62]).

**Conclusion:**

After matching for age, gender and ED diagnosis, we found that patients with an abnormal heart rate, respiratory rate or oxygen saturation at the time of ED arrival or departure are more likely to trigger RRT activation within 12 hours of admission. Early RRT activation was associated with higher mortality at 72 hours and one month, increased rates of invasive intervention and ICU admission. Determining risk factors of early RRT activation is of clinical, operational, and financial importance, as improved medical decision-making regarding disposition would maximize allocation of resources while potentially limiting morbidity and mortality.

## INTRODUCTION

Rapid-response teams (RRTs) (also known as emergency response systems or medical emergency teams) have become an important component of American healthcare and are an integral part of the Institute for Healthcare Improvement’s “Saving 100,000 Lives” campaign.^1^ Hospitals have implemented this concept with the goal of improving clinical outcomes by targeting interventions at patients at risk for clinical deterioration and cardiac arrest.^1^,^2^ These systems have been introduced in many hospitals throughout the world, including our own institution in 2006.

RRTs are interdisciplinary groups that intervene in the care of patients with early warning signs of impending deterioration. The goal of these teams is rapid activation, evaluation and intervention to prevent further clinical deterioration in patients in non-intensive-care settings. 2 In addition to the general concern of care providers, RRT triggers commonly consist of changes in mental status, chest pain, bleeding, and vital sign (VS) deviations.^3^ Despite wide adoption, early data shed some doubt about the effectiveness of RRTs in preventing negative outcomes.^4^ However, recent studies indicate that RRT implementation decreases in-hospital mortality and cardiac arrests,^5^ while the effect of these systems on overall mortality remains unclear.

Safe disposition from the emergency department (ED) is one of the core tasks that emergency physicians are clinically, operationally and socially charged with.^6^ The medical decision-making in selecting admission to a regular floor setting versus an intensive care unit (ICU) is both difficult and high stakes,^7^ particularly considering demonstrated increases in morbidity and mortality in patients admitted to floor settings who deteriorate shortly thereafter.^8^,^9^ Patients with RRT activation within 24 hours of ED admission have been found to have a fourfold increased risk of in-hospital mortality.^10^ It has been suggested that RRT activations, such as these, are often the result of disposition errors, in addition to the scarcity of ICU beds.^11^

Previously, VS based clinical scores have been used to determine the need for rapid evaluation and eventually resuscitative interventions in inpatient settings. The ability of these scoring systems to predict intensive care admission and mortality is controversial,^12^–^15^ and their performance in anticipating deterioration appears to be poor.^14^,^15^ Recently, it has been suggested that the use of RRT criteria as an aid for disposition decisions may be helpful in identifying patients likely to trigger RRT activation and therefore at high risk for morbidity and mortality.^10^

We aim to describe patients having RRT activations within 12 hours of admission from the ED, including clinical characteristics, categorical diagnoses, and outcomes associated to RRT activations and to compare this group with matched controls admitted from the ED who did not activate RRTs. Determining risk factors of early RRT activation is of clinical, operational, and financial importance, as improved medical decision-making regarding disposition would improve allocation of resources while potentially limiting morbidity and mortality.^2^,^7^

## METHODS

### Study Design, Setting and Participants

We obtained institutional review board approval at our institution, and we adhered to the STROBE (Strengthening the Reporting of Observational Studies in Epidemiology) guidelines for reporting observational studies (Appendix A).^16^

We performed a case-control study of patients who presented to an academic tertiary care ED with 73,000 annual patient visits and were admitted to a non-ICU setting. Cases were obtained from an existing prospective, quality-improvement database that collects information regarding all RRT events at our institution, including triggers and outcomes. We included all of the consecutive RRT events recorded from January 2009 to December 2012. Information included in the quality-improvement database was derived from incident reports made by the RRT leader, as well as from the electronic activation system (i.e., pager records).

The RRT is composed of a critical care medicine fellow and attending in critical care medicine, senior level residents, ICU nurses, ICU pharmacists and respiratory therapists. At our institution a RRT is activated if the patient met one of the criteria in Figure. RRT activations were further restricted to only those that occurred within 12 hours of admission from the ED, where the time of admission was the electronic time-stamp of the patient physically leaving the physical plant of the ED (a metric routinely tracked in our ED). Furthermore, in our institution boarding of patients is rare, and time from disposition-decision to the patient leaving the ED is consistently less than two hours.

Controls were patients who presented from January 2011 to December 2012, that were recorded in a routinely maintained, comprehensive patient data warehouse that includes financial, clinical and operations data. From this data warehouse, a randomly selected group of patients who did not trigger a RRT activation within 12 hours of admission was matched to the case group.

Patients included were admitted to the medical or surgical floors. We excluded patients admitted to the ICU. Patients were excluded if they did not consent to having their medical records reviewed for research purposes. Additionally, four patients’ datasets were incomplete and were excluded from the analyses.

### Cases and control identification and data analysis

Patients who had a RRT within 12 hours of admission from the ED from January 2009 to December 2012 were identified from an existing prospective operations and quality-improvement database. The dataset had information on time to RRT activation, reason for activating RRT (primary and secondary criterion), disposition after RRT was called (ICU, no location changed, expired), interventions performed by RRT (intravenous fluids administration, blood transfusion, laboratory work, intubation, etc.), and healthcare provider who activated the RRT (nurse, resident physician, attending physician, other).

Electronic medical records were extracted by a data quality analyst who provided patient demographic information (age, gender), date of ED visit, ED initial complaint, ED final diagnosis, first and last set of VS from the ED visit, disposition from the ED, and time of ED departure. Subsequently, the medical records were reviewed by three of the authors. The following data were extracted into a Microsoft Excel (Microsoft Corporation, Redmond, WA) spreadsheet: mortality within 72 hours and within one month; interventions including dialysis; need for interventions defined as interventional radiology, central line placement, chest tube placement or any operating room procedure. VS and ED diagnoses were manually reviewed for accuracy and consistency.

A convenience sample of 10 charts were abstracted in duplicate by the abstractors, and after standardization of the methods and protocols for the data management each abstractor continued independently on different data sets. Data abstractors met frequently to guarantee quality assurance, maintaining the same methods and protocols.

### Data Analysis

Each control was matched 1:1 to each of the 474 RRT cases by age (+/− 5 years), sex, and ED diagnosis, defined by the final primary ED diagnosis in the record. No secondary diagnosis or comorbidity matching was performed.

VS collected included heart rate, respiratory rate, blood pressure, and oxygen saturation. These were measured and manually entered to the medical record by registered nurses (RN) at the time of the patients’ visits. VS were categorized as the lowest 10%, middle 80%, and highest 10% based on the distribution of all 948 patients included. These cut-offs were selected based on clinical significance of the higher extremes.

Comparisons of categorized VS (first and last heart rate [HR], systolic blood pressure [SBP], O2 Saturation, and respiratory rate [RR] in the ED) between the cases and controls were evaluated using conditional logistic regression models to account for the paired nature of the data. In terms of the timing of the VS records, we defined the first set was defined as the initial group in the record, while the last set was defined consequently as the last group of VS in the record. As described before, in our institution boarding of patients is non-existent; additionally, RNs are mandated to measure and record a set of VS prior to departure from the ED and notify the attending physician if VS are within RRT-activation criteria. Comparisons are reported as odds ratios (OR) with 95% confidence intervals (CI). We did not plan to perform sensitivity or secondary data analyses. Statistical analyses were performed using version 9.3 of the SAS software package (SAS Institute, Cary, NC). All tests were two-sided and p-values <0.05 were considered statistically significant.

## RESULTS

During the study period (January 2009 to December 2012) there were 13,036 RRT activations at our institution. Of these, 1,259 were within 24 hours of admission from the ED (9.7%), and 522 within 12 hours of admission (4.0%).

After removing patients with more than one RRT activation and patients who did not consent for medical record review (<5%), the final studied group comprised 474 cases that had RRT activations within 12 hours of ED admission (Table 1) and 474 age, gender and diagnosis matched controls.

### RRT patients

The mean age of the 474 cases was 65.4 years old, 51.1% males. The most common reasons for activating a RRT were hypotension (21.5%), respiratory distress (18.4%), altered level of consciousness (17.7%), tachycardia (11.2%), oxygen saturation below 90% (9.9%), hypertension (4.2%), chest pain (3.4%), staff concern (2.7%), and seizure (2.3%). The remaining reasons included bradycardia, tachycardia, and arrhythmia and accounted for <9% combined. The care team member calling the RRT was a RN in 70.9%, a physician in 12.7%, and a combination of both a RN/MD in 9.7% of the calls. The caller was unknown 6.8% of the time.

Regarding disposition of the patient following RRT activation, 62.5% were transferred to a higher level of care immediately as an intervention of the RRT and 71.4% were transferred to the ICU within 72 hours. The mortality rate was 4.5% at 72 hours and 17.1% at one month. The percentage of patients requiring an invasive intervention (operating room procedure, interventional radiology, central line placement, chest tube placement, intubation, dialysis, etc.) within 72 hours was 47.6%.

### Comparison RRT cases vs non-RRT matched controls

Odds ratios for the comparison between patients with vs without RRT activation within 12 hours are in Table 2. We also compared disposition between the cases and controls.

VS were evaluated using cut points (lowest 10%, middle 80% and highest 10%) based on the distribution of each VS for all 948 patients in the matched analysis (474 cases and 474 controls combined). Patients who had RRT were more likely to have a first HR recorded in the highest 10% (OR 2.02, 95% CI [1.25–3.27]; were more likely to have a first oxygen saturation recorded in the lowest 10% (OR 2.25, 95% CI [1.42–3.56]); more likely to have a first respiratory rate recorded in the highest 10% (OR 2.92, 95% CI [1.73–4.92]). and had lower oxygen saturations (OR 2.25, 95% CI [1.42–3.56]) upon arrival to the ED.

Additionally, at the time of disposition from the ED to the hospital floor, patients who activated RRT were more likely to have their last HR in the highest 10% percentile (OR 2.76, 95% CI [1.65–4.60]); last systolic blood pressure in the lowest 10% (OR 1.72, 95% CI [1.08–2.72]) and the highest 10% percentile (OR 1.82, 95% CI [1.19–2.80]), last respiratory rate in the highest 10% (OR 4.15, 95% CI [2.44–7.07]); and last oxygen saturation in the lowest 10% percentile (OR 2.29, 95% CI [1.43–3.67]). A detailed description of the VS is depicted in Table 2.

Using VS recorded in the ED and comparing the most extreme 10% measurements on both sides of the distribution (footnote of Table 2), we were able to calculate the values where VS in the ED had a higher likelihood to result in RRT activation within 12 hours of admission. These results showed that the most predictive VS were first and last ED heart rates in the highest 10% (OR 2.02 and 2.76), first and last ED RR in the highest 10% (OR 2.92 and 4.15), and first and last oxygen saturation in the lowest 10% (2.25 and 2.29).

### Outcomes

Patients who triggered RRT activation were more likely to have an ICU admission within 72 hours (OR 38.49, 95% CI [19.03–77.87]), an invasive intervention within 72 hours (OR 5.49, 95% CI [3.82–7.89]), higher mortality at 72 hours (OR 4.24, 95% CI [1.60–11.24]), and higher mortality at one month (OR 4.02, 95% CI [2.44–6.62]).

## DISCUSSION

VS-based clinical scores have been used to predict risk of deterioration in the inpatient setting and the need to escalate interventions,^4^ but until recently the performance of these scores to predict risk of deterioration and RRT activation was not described. Mora et al.^10^ demonstrated recently that ED patients with abnormal VS, particularly heart rate and respiratory rate, are more likely to require RRT activation and had a subsequent higher mortality. This study adds to the recently published evidence, supporting the notion that using a set of predetermined discrete criteria (instead of continuous values as described by Mora et al.) can help identify patients who are at a high risk of RRT activation within 12 hours of admission from an ED. Furthermore, this is one of the largest single-center, case-control datasets of RRT activation published in the literature.

Overall, the proportion of patients admitted through the ED that activate a RRT within 12 hours of admission is very small (4% of all RRT activations at our institution). Therefore, it can be inferred that most patients are admitted to the appropriate level of care and do not decompensate within the first 12 hours. Currently, there is no standard quality metric review analyzing these events, ranging from six (as in our institution) to 24 hours;^17^ therefore, we decided to adopt a 12-hour framework as a pragmatic middle point.

In our population, hypotension and respiratory distress were the most common causes of RRT activation. The mortality rate of patients who triggered early RRT was high, at nearly 5% at 72 hours from admission and 17.5% within 30 days. When compared to non-RRT patients, the odds ratios for mortality were 4.2 at 72 hours and 4.0 at one month, meaning higher mortality in those who activated an RRT; likely this difference in mortality is secondary to the severity of the underlying disease, and despite matching for age, gender and ED diagnosis, it is likely that more variables need to be considered when evaluating this outcome. Our data on mortality is similar to Mora et al.,^10^ in which they found four times greater mortality in patients with RRT activations within 24 hours for ED admission. Mora et al. report a higher respiratory rate and heart rate as the cause for RRT activation and an ICU transfer rate of 20%.^10^ We suspect the difference seen in ICU admissions compared to our study might be secondary to the availability of ICU beds. Supporting this notion, Stelfox et. al^12^ found an association between the number of ICU beds available and ICU admission but no change hospital mortality when the medical emergency team was activated.

There was a significantly increased use of healthcare resources and utilization in the RRT group, as patients who triggered an RRT often required an escalation of care and 42% underwent invasive treatment (dialysis, surgery, central line, chest tube, intubation or interventional radiology procedures) within 72 hours, with an odds ratio of 38.5 for ICU admission and 5.5 for invasive interventions.

RRTs are currently being extensively studied as an integral component of overall patient management systems and procedures.^2^,^10^ Lovett et al. used 24-hour RRTs as a performance improvement tool for emergency physicians in a retrospective analysis fashion.^17^ Similarly, our study adds data to aid in the operational decision of the final disposition of the patient from the ED, providing a cognitive aid for identifying patients who may benefit from a higher level of care. Our results are congruent, albeit with different magnitudes, with recent data from Mora et al.^10^

## LIMITATIONS

First, our study used a prospectively recorded quality dataset for the identification of study subjects, which is susceptible to recording and data-abstractions errors. To mitigate this limitation, we meticulously followed standardized guidelines for observational studies.^16^ Second, when working with medical records, we used data that were recorded for clinical purposes, and therefore some information was missing. Four patients had incomplete data and were excluded from the analyses. Third, our data abstractors were not blinded, and we did not perform statistical measurements of agreement to assess interrater reliability. Fourth, the data of this study were obtained from a single academic center with a complicated patient population and with particular disposition practices. This may limit the generalizability of our findings to other hospitals. Fifth, in matching cases with controls for diagnoses, we were unable to match for secondary diagnoses and comorbidities that may have been helpful in determining potential morbidity and mortality. Sixth, in terms of the RRT activation criteria (Figure), some of its components are not objectively defined (i.e., “A staff member is worried about the patient”); therefore, they depend heavily on training, experience and institutional culture, which further limits generalizability.

## CONCLUSION

After matching for age, gender and ED diagnosis, we found that abnormal heart rate, respiratory rate and oxygen saturation at the time of ED arrival and departure are predictive of RRT activation within 12 hours of admission. Patients who had a RRT activation had higher healthcare utilization with increased ICU admissions, invasive interventions, and mortality at 72 hours and one month.

Among the reasons for activation, tachycardia, hypotension and respiratory distress were the most common. When compared to a population of admitted patients who did not trigger early RRT activation, the early RRT group was more likely to have a diagnosis of hemorrhage, hypotension and sepsis and to have persistent tachycardia, low blood pressure and tachypnea while in the ED. These clinical findings may serve as aids for the identification of patients at risk of deterioration and who therefore may benefit from admission to a higher level of care. Further research is necessary to validate these findings in other practice settings.

Determining risk factors of early RRT activation is of clinical, operational, and financial importance, as improved medical decision-making regarding disposition would improve allocation of resources while potentially limiting morbidity and mortality.

## Supplementary Information



## Figures and Tables

**Figure 1:**
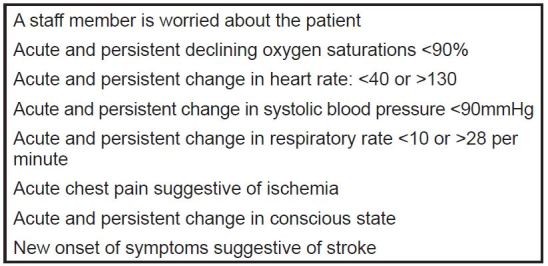
Current clinical criteria used in the study institution for rapid-response team activation.

**Table 1:** Characteristics of 474 patients with rapid-response team (RRT) activation within 12 hours of admission from the emergency department.

Characteristic	Percentages (n=474)
Age +/− standard deviation	65.4 +/− 18.9
Female gender %	48.9%
Reason for activating RRT	
Hypotension	21.5%
Respiratory distress	18.4%
Altered level of consciousness	17.7%
Tachycardia	11.2%
Oxygen saturation below 90%	9.9%
Hypertension	4.2%
Chest pain	3.4%
Staff concern	2.7%
Seizure	2.3%
Other (bradycardia, tachypnea, arrhythmia, etc.)	8.7%
Care team member calling RRT	
Registered nurse (RN)	70.9%
Physician (MD)	12.7%
Combination of RN/MD	9.7%
Unknown	6.8%
Disposition after RRT	
Transferred to higher level of care	62.5%
Transferred to intensive care unit within 72 hours of RRT	71.4%
Mortality	
Mortality within 72 hours of RRT	4.5%
Mortality within 1 month of RRT	17.1%
Intervention (within 72 hours)	
Invasive intervention (operating room, interventional radiology, dialysis, intubation, central line, chest tube, etc.)	47.6%

**Table 2:** Comparisons between patients with and without rapid-response team activations.

Variables	Controls N(%)	Cases N(%)	Odds ratio (95% CI)	P-value*
Disposition	-			
ICU admission (<72h)	-	-	38.49 (19.03–77.87)	<0.001
Intervention (<72h)	-	-	5.49 (3.82–7.89)	<0.001
Mortality (<72h)	-	-	4.24 (1.60–11.24)	0.004
Mortality (<1 month)	-	-	4.02 (2.44–6.62)	<0.001
Vital sign				
First heart rate				
Lowest 10%	52 (11)	38 (8)	0.79 (0.50–1.24)	0.11
Middle 80%	391 (83)	373 (79)	reference	-
Highest 10%	29 (6)	60 (13)	2.02 (1.25–3.27)	0.004
First systolic blood pressure				
Lowest 10%	39 (8)	56 (12)	1.53 (0.98–2.40)	0.062
Middle 80%	389 (83)	365 (77)	reference	-
Highest 10%	42 (9)	50 (11)	1.25 (0.81–1.92)	0.31
First respiratory rate				
Lowest 10%	39 (8)	53 (11)	1.44 (0.91–2.27)	0.12
Middle 80%	405 (86)	353 (75)	reference	-
Highest 10%	26 (6)	65 (14)	2.92 (1.73–4.92)	<0.001
First oxygen saturation				
Lowest 10%	33 (7)	65 (14)	2.25 (1.42–3.56)	<0.001
Middle 80%	388 (83)	352 (75)	reference	-
Highest 10%	49 (10)	54 (12)	1.19 (0.78–1.81)	0.43
Last heart rate				
Lowest 10%	59 (13)	36 (8)	0.61 (0.39–0.96)	0.031
Middle 80%	388 (82)	371 (79)	reference	-
Highest 10%	25 (5)	65 (14)	2.76 (1.65–4.60)	<0.001
Last systolic blood pressure				
Lowest 10%	38 (8)	58 (12)	1.72 (1.08–2.72)	0.022
Middle 80%	393 (83)	351 (75)	reference	-
Highest 10%	40 (8)	62 (13)	1.82 (1.19–2.80)	0.006
Last respiratory rate				
Lowest 10%	51 (11)	44 (9)	0.99 (0.63–1.56)	0.98
Middle 80%	395 (84)	350 (75)	reference	-
Highest 10%	24 (5)	75 (16)	4.15 (2.44–7.07)	<0.001
Last oxygen saturation				
Lowest 10%	31 (7)	61 (13)	2.29 (1.43–3.67)	<0.001
Middle 80%	398 (85)	358 (76)	reference	-
Highest 10%	42 (9)	50 (11)	1.27 (0.82–1.95)	0.29

*ICU*, intensive care unit

* P-value from conditional logistic regression model adjusted for age and sex.

Footnote: Vital signs cut points for vital signs in the bottom 10, middle 80 and top 10% of the distribution are: First heart rate <66, 66–120, >120 per minute; first systolic blood pressure <101, 101–167, >167mmHg, first respiratory rate <16, 16–26, >26 per minute, first oxygen saturation <92, 92–99, >99%, last heart rate <63, 63–112, >112 per minute, last systolic blood pressure <101, 101–157, >157 mmHg, last respiratory rate <16,16–24, >24 per minute, last oxygen saturation <93, 93–99, >99%. These cut points were chosen based on the distribution of each vital sign for all 948 patients.
